# The Protective Effect of Topical PACAP38 in Retinal Morphology and Function of Type 2 Diabetic Retinopathy

**DOI:** 10.3390/ijms26083753

**Published:** 2025-04-16

**Authors:** Lina Li, Evelin Patko, Edina Szabo, Dorottya Molitor, Balazs Meresz, Dora Reglodi, Andras Varga, Diana Denes, Lei Dai, Hongjie Wang, Alexandra Vaczy, Tamas Atlasz

**Affiliations:** 1 Department of Anatomy, HUN-REN-PTE PACAP Research Team, Centre for Neuroscience, University of Pecs Medical School, Szigeti Str. 12, H-7624 Pecs, Hungary; linali1124259963@gmail.com (L.L.); evelin.patko@gmail.com (E.P.); szaboedina90@gmail.com (E.S.); molittty@gmail.com (D.M.); mereszbalazs@gmail.com (B.M.); dora.reglodi@aok.pte.hu (D.R.); varga.andras@aok.pte.hu (A.V.); denesdiana@gmail.com (D.D.); alexandra.vaczy@aok.pte.hu (A.V.); 2 Division of Cardiology, Department of Internal Medicine, Tongji Hospital, Tongji Medical College, Huazhong University of Science and Technology, Wuhan 430030, China; dailei@tjh.tjmu.edu.cn (L.D.); hongjie.wang@tjh.tjmu.edu.cn (H.W.); 3 Hubei Provincial Engineering Research Center of Vascular Interventional Therapy, Wuhan 430030, China; 4 Department of Sports Biology and Kinesiology, University of Pecs, Ifjusag Str. 6, H-7624 Pecs, Hungary

**Keywords:** type 2 diabetic retinal disease, PACAP, eye drops, OCT, ERG

## Abstract

The continuously growing diabetes population is a significant concern with type 2 diabetic retinal disease (T2DRD), which is a leading cause of permanent blindness. However, the underlying pathophysiological mechanism of T2DRD has not yet been fully understood. Pituitary adenylate cyclase-activating polypeptide (PACAP) was first isolated from the ovine hypothalamus based on its stimulating effect on the adenylate cyclase enzyme in anterior pituitary cells. PACAP38 (PACAP with 38 amino acids) activates anti-apoptotic pathways, inhibits pro-apoptotic signaling, and creates an anti-inflammatory environment in the retina. The aim of the present study was to test the possible retinoprotective effect of the topical administration of PACAP38 in a type 2 diabetic animal model induced by a high-fat diet and the intraperitoneally injected low-dose streptozotocin (STZ). Wistar rats were divided into four groups: the control, control + PACAP38, diabetes, and diabetes + PACAP38 groups randomly. Type 2 diabetes was induced with the combination of STZ (30 mg/kg) and a high-fat diet. All rats were treated topically two times a day for 16 weeks: the control + PACAP38 and diabetes + PACAP38 groups were applied with PACAP38 eye drops (1 µg/drop), while the control and diabetes groups were administered using vehicles (artificial tears). The diabetes model was validated by a fasting oral glucose tolerance test (OGTT) and C-peptide ELISA test. Animals were monitored during the whole experiment for the progression of the disease using electroretinography (ERG) and optical coherence tomography (OCT). Post-mortem immunohistochemistry and a vessel analysis were performed in the retina samples after 16 weeks. An OGTT, a C-peptide ELISA test, and the investigation of blood parameters proved the development of type 2 diabetes. Significant differences could be detected in visual function between the two diabetic groups at week 16 (in the a-wave, b-wave, and OP amplitudes), where the diabetes PACAP38-treated group was similar to the control ones. OCT measurements correlated with ERG data, where the total retinal thickness was preserved in the diabetes + PACAP38 group. PACAP38 also protected the microvascular structure in the retina. Topically administered PACAP38 has potent neuroprotective effects against type 2 diabetic retinal disease; therefore, it could be a promising therapeutic approach for the treatment of T2DRD.

## 1. Introduction

Type 2 diabetes is a form of diabetes mellitus characterized by high blood glucose levels, insulin resistance, and a relative insulin deficiency. The most common but initially occult complication in diabetic people, diabetic retinopathy (DR), has been recognized as the leading cause of permanent blindness in the continuously growing diabetic population [1]. Although DR has traditionally been classified as a microvascular disease, recent studies have found that neurosensory destruction is also a significant factor in its pathophysiology [2,3,4,5].

Several animal models of type 2 diabetes exist for examining the mechanisms of diabetic complications, including non-obese spontaneously polygenic Goto-Kakizaki animals, Nile rats, or Zucker diabetic fatty (ZDF) rats [6,7,8,9]. Moreover, a high-fat-fed/streptozotocin (STZ)-induced hyperglycemic rat model is now being used for investigating the neural and pharmacologic complications in the eye. Davidson and coworkers found that menhaden oil, α-lipoic acid, or enalapril or their combination can be used to treat type 2 diabetic rats that have chronic hyperlipidemia and hyperglycemia, which can result in corneal nerve regeneration and sensitivity recovery [10]. Others revealed that topical (eye drops) treatment of somatostatin prevents retinal neurodegeneration in C57BLKS/J db/db mice, a spontaneous model of type 2 diabetes, induced by STZ [11]. Moreover, some cohort studies concerning type 1 DR found more retinal morphological abnormalities, such as irregular thinning areas like the foveal center [12] and significant total retinal thickness changes due to the diminution of the inner retinal layers [13]. One of the latest preclinical animal studies has documented the thinning of the ganglion and photoreceptor cell layers [14]. Cogan et al. separated the retinal vessel architecture from the neurosensory retina by the trypsin digestion method and emphasized the pericyte loss in post-mortem eyes [15]. Subsequent studies detected more pathological changes like microaneurysms, endothelial proliferation, acellularity, strand formation [16], and microvascular lesions during the early stage of diabetic retinal disease in rat retinas [17]. A recent study of human samples has provided strong evidence that superficial vessel density is correlated with neural impairments evaluated by ERG [18]. Another large sample size of human eyes affected by diabetic retinal disease suggested further that impaired retinal capillary perfusion was dependent on the differentiated leaking condition associated closely with microaneurysm size [19].

Pituitary adenylate cyclase-activating polypeptide (PACAP) was first isolated from the ovine hypothalamus based on its stimulating effect on the adenylate cyclase enzyme in anterior pituitary cells [20]. PACAP has two isoforms, PACAP27 and PACAP38, with 27 and 38 amino acids, respectively. PACAP activates anti-apoptotic pathways, inhibits pro-apoptotic signaling pathways, and creates an anti-inflammatory environment in the retina [21,22,23]. In vivo, the protective effects of intravitreally or topically administered PACAP38 have been shown by our research group in different rat or mouse models of retinopathies, such as excitotoxic injury induced by glutamate [24,25], ischemic injury induced by carotid artery ligation [26], degeneration caused by UV-A light [27], LPS-induced inflammation [23], microbead-induced glaucoma [28], and streptozotocin-induced type 1 DR [29]. Postyeni et al. showed the protective role of PARP inhibitor Olaparib and PACAP double treatment in hypertensive diabetic rat retinas [30]. In 2021, D’Amico and coworkers summarized the data on the protective role played by PACAP and an active eight-amino acid neuroprotective snippet (NAP) in the retina. They provided an overview of the association between PACAP and activity-dependent neuroprotective protein (ADNP) in DR [31]. They have recently shown that the PACAP-ADNP axis prevents the outer retinal barrier breakdown and choroidal neovascularization in DR [32]. Despite all these data, we have no evidence regarding the potential role of anti-inflammatory effects of non-invasive, PACAP38 eye-drop therapy in a widely used type 2 diabetes model. Therefore, the aim of the present study was to test the possible protective effect of the topical administration of PACAP38 in a type 2 diabetic animal model induced by a high-fat diet and the intraperitoneally injected STZ.

## 2. Results

### 2.1. Glucose Response, Fasting Glucose Levels, and Hypertriglyceridemia Following PACAP Administration

**Figure 1:**
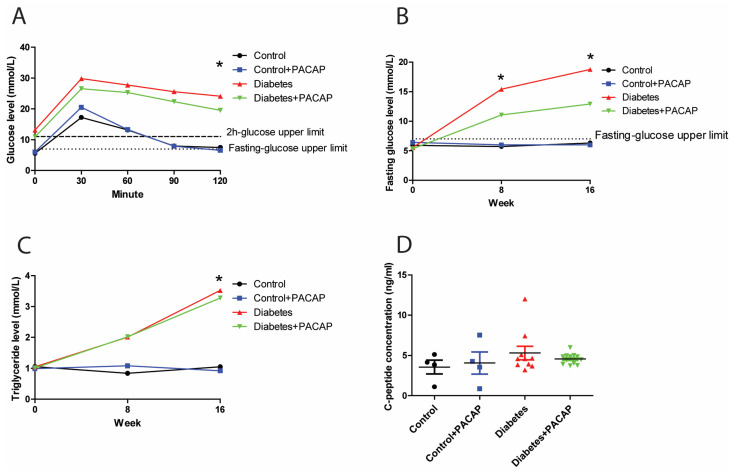
Effects of PACAP treatment on blood sugar response, fasting glucose levels, and high triglycerides in type 2 diabetes. (**A**) Recording of glucose tolerance test (intraperitoneal) 8 weeks after streptozotocin (STZ). Both the diabetes and diabetes + PACAP groups exhibited a significant rise in glucose levels compared to the control group (diabetes: * *p* = 0.005, diabetes + PACAP: * *p* = 0.004. The lower dashed line represents the normal fasting glucose level (7.8 mmol/L), while the upper dashed line indicates the 2-hour glucose threshold (11.1 mmol/L). (**B**) Longitudinal analysis of fasting glucose levels. Diabetic rats displayed a progressive elevation in glucose levels compared to the control group (8th weeks: * *p* = 0.004, 16th weeks: * *p* = 0.004). The dashed line represents the fasting glucose level (7.8 mmol/L). (**C**) Longitudinal analysis of triglyceride levels. The diabetes and diabetes + PACAP groups showed a significant elevation in triglyceride levels compared to the control group (diabetes: * *p* = 0.033, diabetes + PACAP: * *p* = 0.023). The dashed line represents the normal triglyceride level (0.79 mmol/L). (**D**) Analysis of C-peptide levels in control, control + PACAP, diabetes, and diabetes + PACAP groups after 8 weeks of STZ treatment. No statistical significance was observed between the groups. Significant difference compared to the control group (* *p* < 0.05). Abbreviations: PACAP: pituitary adenylate cyclase-activating polypeptide.

In the intraperitoneal glucose tolerance test, the 2 h glucose value of rats with diabetes (diabetes group and diabetes + PACAP group) was three times higher than in control rats (24.12 ± 2.2 mmol/L (diabetes) vs. 7.48 ± 0.31 mmol/L (control); 19.55 ± 2.32 mmol/L diabetes + PACAP) vs. 6.58 ± 0.34 mmol/L (control + PACAP)) (Figure 1A). Similarly, the fasting glucose level in the disease group exhibited a continuously elevating trend compared to the stability in normal ones. In the 8-week diabetic samples, the fasting glucose level was elevated to 15.43 ± 1.9 mmol/L (diabetes) vs. 5.73 ± 0.15 mmol/L in the control group, and, in the diabetes + PACAP group, to 11.05 ± 1.19 mmol/L, vs. 6 ± 0.27 mmol/L in the control + PACAP group. There is no significant difference between the diabetes and the diabetes + PACAP group at both the eighth and sixteenth week. (Figure 1B). At week 16, blood glucose levels showed a further increase in the diabetic groups compared to non-diabetic ones. Meanwhile, triglyceride levels were in line with the findings of the fasting glucose level (Figure 1C). Both the diabetes group and diabetes + PACAP group had significantly higher serum triglycerides than the control ones (3.52 ± 0.76 mmol/L (diabetes) vs. 1.05 ± 0.12 mmol/L (control); 3.28 ± 0.52 mmol/L (diabetes + PACAP) vs. 0.92 ± 0.05 mmol/L (control + PACAP). The C-peptide immunoassay, which confirmed the presence of the type 2 diabetes model, indicated that there was no statistical difference between the disease and normal ones (Figure 1D).

### 2.2. Protective Effect of PACAP Eye Drops on Visual Responses

**Figure 2:**
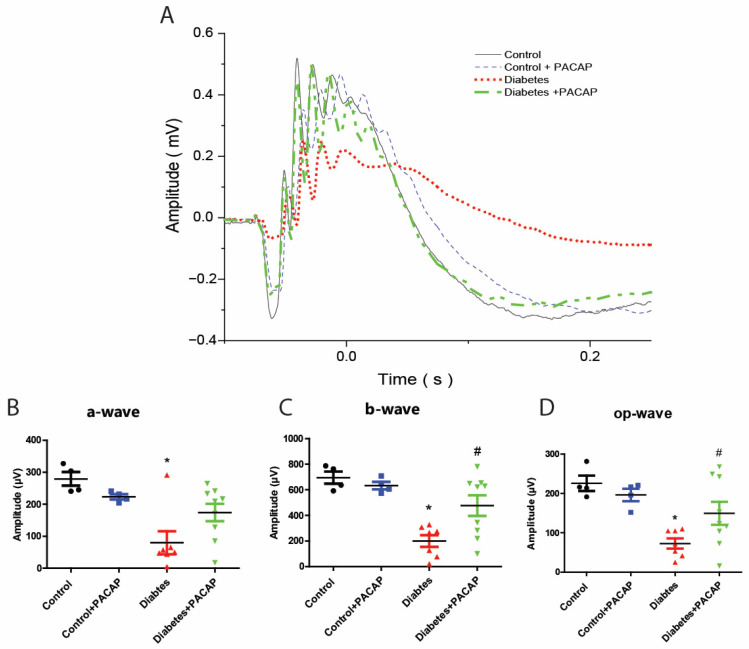
Protective effect of PACAP eye drops on vision. (**A**) Representative recordings of scotopic ERG in the control, control + PACAP, diabetes, and diabetes + PACAP groups at the endpoint of the experiment. (**B**) Analysis of a-wave amplitude. Diabetic rats exhibited a significant reduction in a-wave amplitude compared to controls (* *p* = 0.023). (**C**) Analysis of b-wave amplitude. Diabetic rats showed statistical significance in b-wave amplitude compared to control (* *p* < 0.001, # *p* = 0.015). (**D**) Investigation of oscillatory potential wave amplitude also revealed statistical significance between diabetes and control (* *p* < 0.001), and diabetes and diabetes + PACAP (# *p* = 0.045). *: significant difference compared to the control group; #: significant difference compared to the diabetes group (*p* < 0.05). Abbreviations: OP: oscillatory potential; PACAP: pituitary adenylate cyclase-activating polypeptide.

As the most sensitive marker of retinopathy preceding the onset of diabetic retinal disease [33], our electroretinogram has documented that diabetic rats had severe functional damage (Figure 2A). The amplitude of the a-wave was significantly decreased (79.9 ± 13.59 µV (diabetes) vs. 279.24 ± 10.74 µV (control)), and also that of the b-wave (200.01 ± 17.55 µV (diabetes) vs. 695.02 ± 23.76 µV (control)), and oscillatory potential (OP) waves (72.99 ± 4.89 µV (diabetes) vs. 225.81 ± 9.73 µV (control)) (Figure 2B–D).

In contrast to diabetic rats, the diabetes + PACAP group had a more sensitive response in all amplitudes, like the a-wave (79.9 ± 13.59 µV (diabetes) vs. 174.23 ± 9.12 µV (diabetes + PACAP)), b-wave (200.08 ± 17.55 µV (diabetes) vs. 476.05 ± 26.69 µV (diabetes + PACAP)), and oscillatory potential waves (72.99 ± 4.89 µV (diabetes) vs. 149.75 ± 9.64 µV (diabetes + PACAP)) (Figure 2B–D).

### 2.3. Effects of PACAP Eye Drop Treatment on Histological Changes

**Figure 3:**
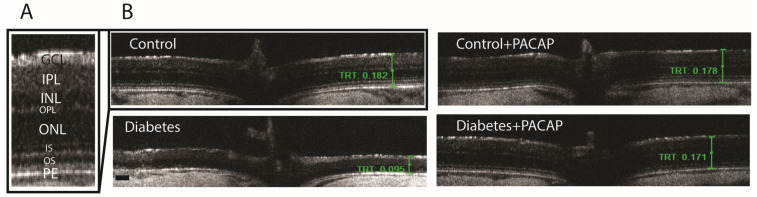
Histological effects of PACAP eye drops treatment. Representative OCT images comparing retinal thickness 16 weeks after STZ injection. (**A**) Magnified view of the retinal structure highlighting the different histological layers. (**B**) Representative cross-sectional B-scans from the control, control + PACAP, diabetes, and diabetes + PACAP groups, showing retinal thickness (mm). Abbreviations: PACAP: pituitary adenylate cyclase-activating polypeptide, GCL: ganglion cell layer; IPL: inner plexiform layer; INL: inner nuclear layer; OPL: outer plexiform layer; ONL: outer nuclear layer; IS: inner segment; OS: outer segment; PE: pigment epithelium; TRT: total retinal thickness. Scale bar: 100 µm.

**Figure 4:**
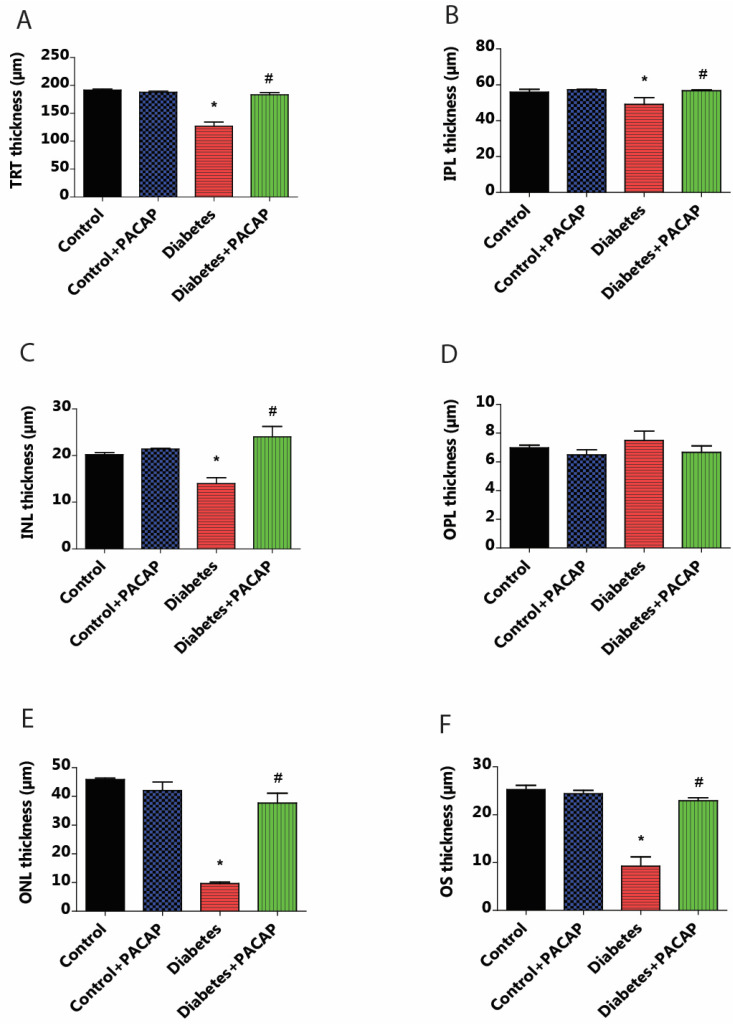
Effects of PACAP eye drop treatment on retinal layer thicknesses. Statistical analysis of retinal thickness was performed using OCT B-scan images 16 weeks after STZ injection. Diabetes resulted in a significant reduction in total retinal thickness (TRT) compared to the control group (* *p* < 0.001). However, the diabetes + PACAP group exhibited significantly thicker TRT than the diabetes group (# *p* < 0.001) (**A**). Diabetes showed significant thinning retinal layers compared to the control group in the outer segment (OS) (* *p* < 0.001) (**F**), outer nuclear layer (ONL) (* *p* = 0.02) (**E**), inner nuclear layer (INL) (* *p* = 0.02) (**C**), and in the inner plexiform layer (IPL) (* *p* = 0.005) (**B**). In contrast, the diabetes + PACAP group demonstrated significantly greater thickness than the diabetes group in OS (# *p* < 0.001) (**F**), ONL (# *p* = 0.005) (**E**), and INL (# *p* = 0.005) (**C**). Interestingly, the outer plexiform layer (OPL) in the diabetic group appeared potentially thicker than both the control and diabetes + PACAP groups (**D**). *: vs. control; #: vs. diabetes group (*p* < 0.05). Abbreviations: PACAP: pituitary adenylate cyclase-activating polypeptide, TRT: total retinal thickness; IPL: inner plexiform layer; INL: inner nuclear layer; OPL: outer plexiform layer; ONL: outer nuclear layer; OS: outer segment.

OCT indicated that diabetes has an apparently degenerative effect on the retina (Figure 3B) compared to normal conditions (Figure 3A,B). Our results at the endpoint showed a significant reduction in total retinal thickness (TRT) to normal thickness (127.05 ± 6.94 µm (diabetes) vs. 191.51 ± 1.93 µm (control)). This decrease was further supplemented by the individual layer comparison with controls, such as the inner nuclear layer (INL) thickness (13.95 ± 1.3 µm (diabetes) vs. 20.19 ± 0.49 µm (control)), outer nuclear layer (ONL) thickness (9.65 ± 0.52 µm (diabetes) vs. 45.85 ± 0.51 µm (control)), and outer segment layer (OS) thickness (9.22 ± 1.95 µm (diabetes) vs. 25.27 ± 0.86 µm (control)) (Figure 4A–F).

As compared to the diabetes group, the diabetes + PACAP group presented a different phenomenon: it was 50% thicker in TRT (127.05 ± 6.94 µm (diabetes) vs. 182.97 ± 4.15 µm (diabetes + PACAP)); there were also significantly higher values in the majority of nuclear layers, such as the INL thickness (13.95 ± 1.3 µm (diabetes) vs. 24.03 ± 2.22 µm (diabetes + PACAP)), ONL thickness (9.65 ± 0.52 µm (diabetes) vs. 37.67 ± 3.43 µm (diabetes + PACAP)), and OS thickness (9.22 ± 1.95 µm (diabetes) vs. 22.91 ± 0.65 µm (diabetes + PACAP)). Moreover, the inner plexiform layer (IPL) showed a significant difference (49.01 ± 3.91 µm (diabetes) vs. 56.63 ± 0.56 µm (diabetes + PACAP)). Interestingly, the outer plexiform layer (OPL) in the diabetic group (7.5 ± 0.65 µm) showed no significant difference compared to control (6.98 ± 0.18 µm) (Figure 4A–F).

### 2.4. Protective Effects of PACAP on Retinal Vasculature

**Figure 5:**
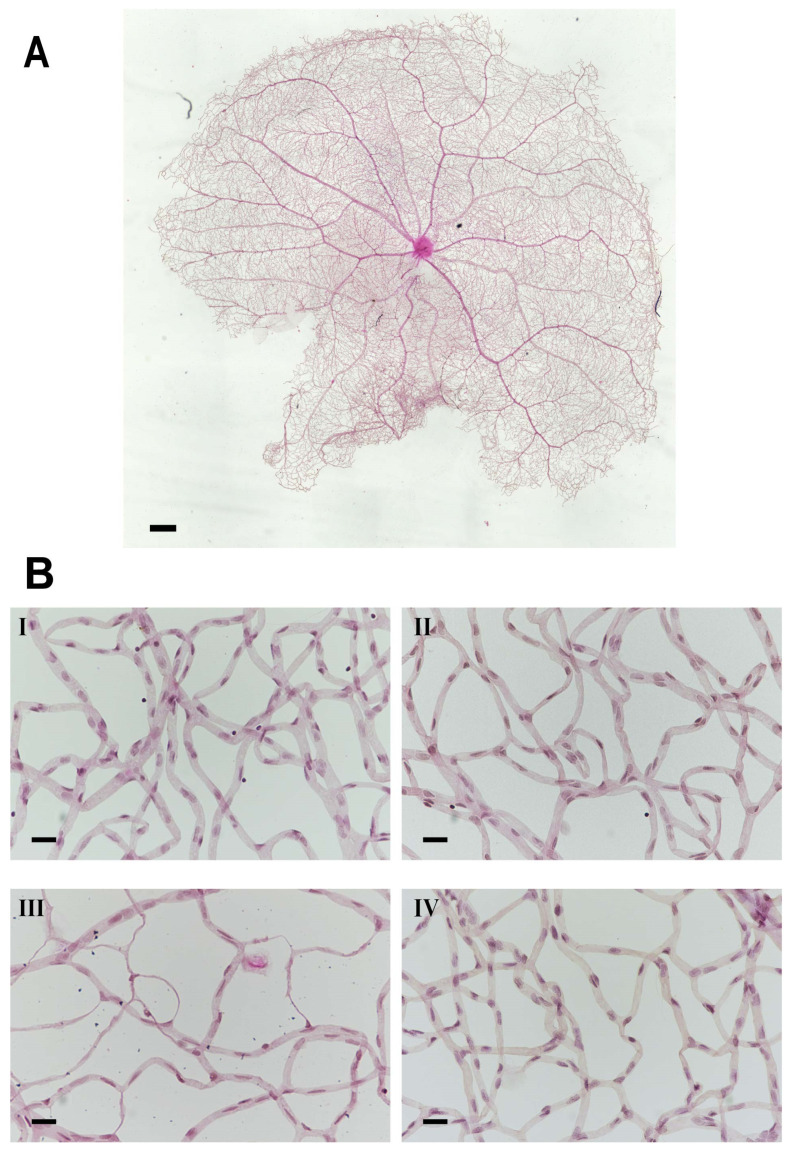
Microvascular histopathology after PACAP treatment. (**A**) Normal whole retinal vessel architecture in rats. (**B**) Representative images of the retinal microvascular system in the four groups ((**I**): control; (**II**): control + PACAP; (**III**): diabetes; and (**IV**): diabetes + PACAP), using the retina-trypsin digestion flat-mount approach and stained with hematoxylin and eosin. Scale bar: 500 µm (**A**), and 100 µm (**B**).

**Figure 6:**
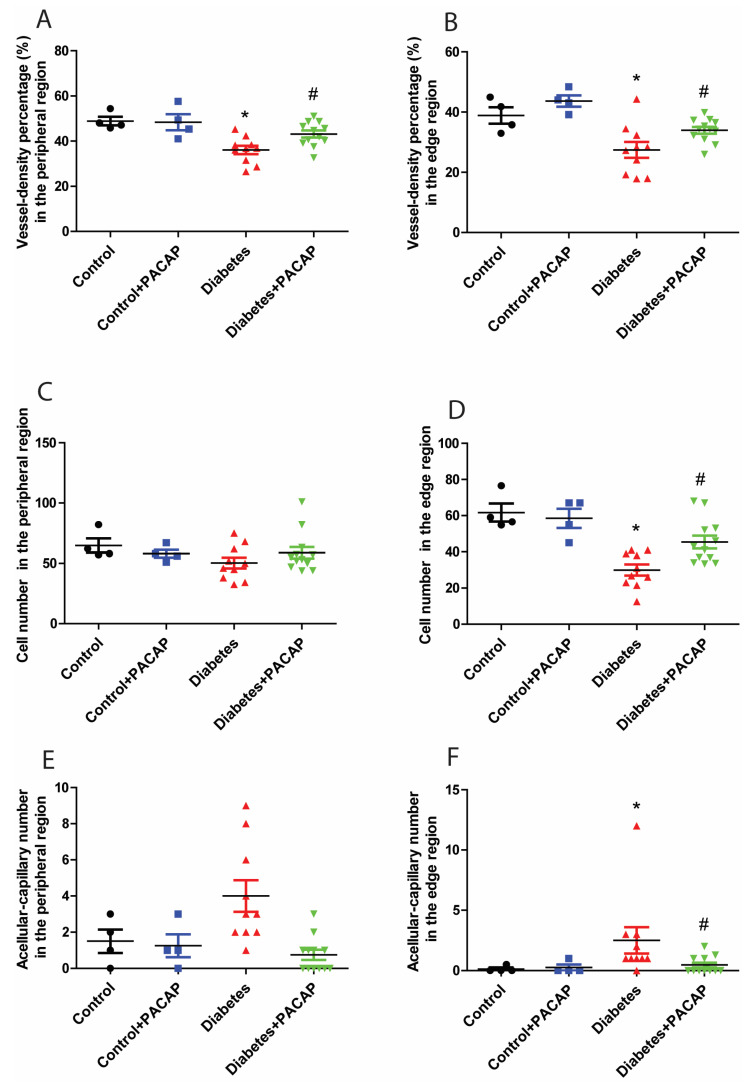
Impact of PACAP treatment on microvascular histopathology. Analysis of the vessel density (VD), and number of pericytes and acellular capillaries (ACs). Diabetic retinas exhibited a significant decrease in VD in the peripheral (* *p* = 0.002) (**A**) and edge (* *p* = 0.003) (**B**) regions compared to the control group. However, PACAP-treated diabetic retinas showed a statistically significant increase in VD (peripheral: # *p* = 0.008; edge: # *p* = 0.02). Regarding the number of pericytes, significant differences were observed between the disease and control group in the edge region (* *p* = 0.005) (**D**), but not in the peripheral region (*p* > 0.05) (**C**). In contrast, diabetes was associated with a significantly higher number of acellular capillaries compared to the control group in the edge region (* *p* = 0.01) (**F**), but not in the peripheral region (*p* > 0.05) (**E**). Notably, the diabetes + PACAP group demonstrated a substantially lower number of AC compared to the diabetes group in the edge region (# *p* = 0.01) (**F**). Symbols represent the number of elements in the different groups investigated. *: vs. control; #: vs. diabetes group (*p* < 0.05). Abbreviations: PACAP: pituitary adenylate cyclase-activating polypeptide.

Retina trypsin digestion flat mounting is a critical approach toward visualizing the retinal vasculature, especially microvascular histopathology (Figure 5A,B). In line with previous research work, several signs regarding microvascular lesions were recorded in our study (Figure 5B). Our observation showed that the percentage of vessel density from the diabetic retinal vessel architecture became less in the peripheral area (36.08 ± 1.83% (diabetes) vs. 48.85 ± 1.90% (control)) (Figure 6A), and dramatically dropped in the edge region (27.43 ± 2.62% (diabetes) vs. 38.89 ± 2.75% control)) (Figure 6B). Correspondingly, pericytes from the diabetes group were also slightly affected in the periphery (50.28 ± 4.51 (diabetes) vs. 64.8 ± 5.83 (control) (Figure 6C), but statistically reduced in the edge regions (29.98 ± 3.06 (diabetes) vs. 61.71 ± 5.00 (control) (Figure 6D)). The number of acellular capillaries in T2DRD did not differ from that in the controls in the peripheral area (4.00 ± 0.27 (diabetes) vs. 1.50 ± 0.32 (control) (Figure 6E). However, a significantly higher number of capillaries were detected in diabetic retinas compared to normal retinas in the edge region (2.50 ± 0.34 (diabetes) vs. 0.125 ± 0.06 (control)) (Figure 6F).

In contrast to the diabetes group, the percentage of vessel density from the diabetes + PACAP group presented higher in the peripheral area (36.08 ± 1.83% (diabetes) vs. 43.13 ± 1.55% (diabetes + PACAP)) and the edge region (27.43 ± 2.62% (diabetes) vs. 33.96 ± 1.12% (diabetes + PACAP)) (Figure 6A,B). Meanwhile, the number of pericytes in the diabetes + PACAP group also exhibited higher survival rates in both areas (periphery: 50.28 ± 4.51 (diabetes) vs. 60.12 ± 4.84 (diabetes + PACAP); edge: 29.98 ± 3.06 (diabetes) vs. 46.51 ± 3.54 (diabetes + PACAP)) (Figure 6C,D). In contrast, under diabetic circumstances, in acellular capillaries, no changes were detected in the PACAP-treated group (0.75 ± 0.08) than in the diabetes group (4.00 ± 0.27) in the peripheral area, while significance could be observed in the edge region (2.50 ± 0.35 (diabetes) vs. 0.46 ± 0.07 (diabetes + PACAP)) (Figure 6E,F).

## 3. Discussion

Our study demonstrated that type 2 diabetic retinal dysfunction (T2DRD) precedes observable vascular deterioration, as evidenced by various methodologies, including functional assessments via ERG, structural analysis using OCT, microvascular investigations, and immunohistochemistry. Based on the comprehensive dataset, our novel findings provide the first evidence that metabolic symptoms are correlated with the progression of diabetic retinal dysfunction, consistent with the observations of Karaca et al. [34].

Even under conditions of severe metabolic disorder, our results showed that PACAP38 exhibits a strong protective effect on the retina, effectively mitigating the impact of type 2 diabetes on retinal function and structure.

As a sensitive method for detecting early retinopathy [35], the electroretinogram (ERG) provides valuable insights into visual function, as the amplitude, implicit time, and latency of the scotopic threshold response are significantly correlated with the severity of DR [36]. Additionally, scotopic ERG is particularly effective in assessing rod-dominated retinal function in response to light stimuli [33]. In our study on type 2 diabetes, scotopic ERG revealed that all amplitudes began to decrease as early as the first month. A structural ocular analysis further confirmed abnormal retinal thinning by the study’s endpoint, consistent with the findings of Ahmed and El-Mansi [37]. Moreover, the inner retinal thinning observed aligns with the results reported by Sohn et al. [3]. Our studies revealed that the corneal negative a-wave amplitude, predominantly reflecting rod photoreceptor activity, was reduced in diabetic control samples. This finding, consistent with the ERG results, was further supported by OCT analysis, which demonstrated a significant decline in both ONL and OS thickness due to diabetes. These observations align with previous findings by El-Mansi [37], indicating that photoreceptor cells and their outer segments undergo severe deterioration under metabolic disorders. Numerous studies suggest that the photoreceptor layer is highly sensitive to alterations in the retinal biochemical metabolism [9,38]. In our study, the ONL thickness began to decline, accompanied by a reduction in the a-wave amplitude, after 16 weeks of exposure to hyperglycemia and hyperlipidemia. However, treatment with PACAP38 provided significant protection, preserving ONL and OS thickness throughout the experiment. Compared to the a-wave, numerous photopic ERG studies have demonstrated that a reduction in the b-wave amplitude and a delay in peak time are among the most significant indicators of diabetic retinopathy [39,40]. Our ERG analysis also showed a notable reduction in b-wave amplitude in the beginning of the second month of diabetes and a slowing of the response velocity at the endpoint of the study, suggesting a severe impairment of rod-driven bipolar cell function. Correspondingly, our OCT analysis revealed abnormal thinning of the INL and a progressive reduction in IPL thickness in the diabetic group, providing structural evidence to complement the observed retinal dysfunction. In contrast, diabetic retinas treated with PACAP38 exhibited more responsive ERG patterns and reduced structural abnormalities in both the INL and IPL, indicating a retinoprotective effect. Dark-adapted oscillatory potentials (OPs), covered on the ascending limb of the b-wave [35], have been recognized as one of the earliest signs of diabetic retinopathy, as established by numerous studies [41,42]. While the exact cellular origin of OPs remains uncertain, they reflect inner retinal activity, particularly involving amacrine [43] and ganglion cells [33,44]. Previous ERG studies on diabetic retinopathy have consistently reported a reduction in OP amplitude and an increase in peak time [41,42], highlighting the diagnostic value of OP alterations for the early detection of diabetic retinopathy [35]. Our findings confirmed a significant reduction in the OP wave amplitude caused by diabetes, likely due to weaker signal transmission through the IPL, as supported by our OCT results. Additionally, we demonstrated that treatment with PACAP38 provided both functional and structural protection to the inner retina, moderating diabetes-induced damage.

Functional and structural impairments were observed in the diabetic group without any detectable vascular alterations on fundoscopy images. Kim and colleagues reported that neural impairment is closely linked to vascular dysfunction in type 2 diabetic retinas [18]. In addition to microaneurysms and pericyte ghost cells, our study found that diabetic rats exhibiting poor performance in scotopic ERG and OCT analysis were at a higher risk of significant microvascular abnormalities. These included a reduced vessel density, a greater number of acellular capillaries, and substantial pericyte loss [15,16], particularly at the peripheral edges of the retinal vasculature. A notable feature of diabetic microvascular pathology is that degeneration begins at the edges of the vasculature and progresses to the peripheral regions, with the central retina showing greater resilience to diabetes-induced damage. In contrast, PACAP38 demonstrated a strong protective effect against these microvascular lesions. Additionally, lymphocyte accumulation was observed within the diabetic vessel networks, likely associated with elevated expression levels of ICAM-1, which promotes leukocyte trapping in capillaries [45,46]. This accumulation suggests an increased capillary permeability, as indicated by the lymphocyte efflux from the retinal vessels.

Most of the cytoprotective effects of PACAP are mediated through the activation of the PAC1 receptor, which induces a signaling cascade to stimulate protective factors and inhibit caspase activation [47]. Our previous studies using a type 1 streptozotocin (STZ)-induced diabetic rat model demonstrated that intravitreal PACAP administration significantly mitigated retinal structural damage. PACAP treatment effectively reduced neuronal cell loss in the ganglion cell layer (GCL), preserved cone cell integrity, and maintained dopaminergic amacrine cell numbers, reinforcing its strong neuroprotective potential in diabetic retinopathy (DR). We observed that PACAP treatment upregulates PAC1-R expression in the retina, sometimes even in cells where PAC1-Rs are normally absent [48,49,50]. Additionally, PACAP plays a key role in modulating inflammatory responses, as it has been shown to reduce IL-1β expression and downregulate VEGF and VEGFRs in STZ-treated animals [51]. These effects contribute to blood–retinal barrier protection and the prevention of DR progression [50,52]. Furthermore, in our previous study, we demonstrated that PACAP treatment alleviates retinal injury by regulating key apoptotic pathways. PACAP administration increased the expression of anti-apoptotic proteins, including p-Akt, extracellular signal-regulated kinase (p-ERK1/2), PKC, and Bcl-2, while simultaneously reducing the levels of pro-apoptotic markers, such as phosphorylated p38MAPK and activated caspase−3, −8, and −12. Moreover, we confirmed that PACAP exerts its protective effects through key molecular signaling pathways, particularly cAMP/PKA and PI3K/Akt, both of which are crucial for reducing apoptosis and promoting cell survival [49,50]. Endogenous PACAP has also been shown to exert protective effects. Studies have demonstrated that mice lacking endogenous PACAP are more vulnerable to injuries, including retinal ischemia [48,53]. Additionally, PACAP and its specific PAC1-R have been reported to be upregulated following various types of injuries [54]. Tsutsumi et al. described that the activation of the VPAC1 receptor is associated with increased glucose output, while the activation of the VPAC2 receptor may play a role in insulin secretion [55]. Therefore, all three PACAP receptors may contribute positively to combating diabetes and its complications. Based on these findings, we propose that PACAP could be highly effective in diabetic retinopathy through the same signaling mechanisms observed in the type 1 STZ model when administered via intravitreal injection. To further clarify the precise molecular pathways involved in PACAP’s protective effects, we plan to conduct a transcriptome analysis in the near future to identify the critical signaling cascades that mediate its therapeutic benefits in type 2 DR.

## 4. Materials and Methods

### 4.1. Animals

Two-month-old male Wistar rats weighing 305.57 g ± 23.43 g were used in our experiments. Animals were maintained in the animal house that is humidity-controlled and has an ambient temperature of 21 ℃, subjected to a 12 h light/dark cycle, and fed and watered ad libitum. The institutional guidelines approved all animal procedures (BA02/2000-02/2022, University of Pecs).

These rats were divided randomly into four groups: (i) control (*n* = 4), (ii) control + PACAP (*n* = 4), (iii) diabetes (*n* = 10), and (iv) diabetes + PACAP (*n* = 12). The rats in diabetes and diabetes + PACAP groups were injected with Streptozotocin (STZ) intraperitoneally twice (one week interval) (30 mg/kg, i.p.) and fed with high-fat-diet food (fat 45%, protein 18%, and carbohydrate 37%, Altromin, Lage, Germany) at the same time to induce diabetes initiation, as described by Guo and Diego [56,57]. Control groups were fed with regular chows.

The control + PACAP and diabetes + PACAP groups administered PACAP38 eye drops (1 µg/drop, 1 drop/eye) twice a day. PACAP38 was synthesized by the Department of Medical Chemistry, University of Szeged, Szeged, Hungary, and was dissolved in artificial tears (Systane, Alcon, Budapest, Hungary). PACAP38 was replaced with artificial tears in the control and diabetes groups. Rats were treated twice daily with one drop for four consecutive weeks. Sixteen weeks after the STZ injection, all animals were killed with overdose anesthesia (Isoflurane, Baxter, Budapest, Hungary).

### 4.2. Glucose Tolerance Test, Fasting Glucose, and Triglycerides Levels

Rats were fasted overnight (8 h), and blood-fasting glucose from the tail vein was measured. Animals were injected with 40% glucose dissolved in 0.1 M phosphate-buffer saline (PBS) (pH = 7.4) intraperitoneally (2 g/b.w. kg), and then we measured the glucose level at 0, 30, 60, 90, and 120 min after the glucose injection. Triglyceride level was also determined by triglyceride strips (Roche, Budapest, Hungary). All parameters were monitored at day 0, and on the 8th and 16th weeks. Rats with fasting glucose levels higher than 7.1 mmol/L and 2 h glucose values of oral glucose tolerance test higher than 11.1 mmol/L were excluded from further experiments.

### 4.3. C-Peptide Enzyme Immunoassay

C-peptide enzyme immunoassay was employed to distinguish type 2 from type 1 diabetic rats. Rats were fasted overnight (8 h) during the eighth week of diabetic duration. Blood samples were collected from the rat’s tail vein under inhalational anesthesia. The collected blood sample was mixed homogeneously with 7.5% EDTA and centrifuged at 2200 rpm for 15 min at 4 °C. Only serum was collected and stored at −20 °C until use. RayBio^®^ Rat C-peptide immunoassay kit (RayBio^®^ Rat, RayBiotech, Peachtree Corners, GA, USA) was employed to determine the C-peptide enzyme level. All experimental procedures were followed by the user manual of manufacture [58]. C-peptide in serum was measured spectrophotometrically at 450 nm by an ELISA reader (Biotek EL800, Winooski, VT, USA).

### 4.4. Spectral-Domain Optical Coherence Tomography (SD-OCT)

SD-OCT studies, an advanced, non-invasive, and productive way to provide high-resolution morphological details of ocular structure in real time, were tracked before the experiment (day 0) and at the 8th and 16th weeks. Rats were anesthetized by ketamine 5% (*w*/*v*, Calypsol, Richter Gedeon, Budapest, Hungary, 90 mg/b.w. kg, i.p.), together with xylazine 20% (*w*/*v*, Sedaxylan, Dechra, The Netherlands, 10 mg/b.w. kg, i.p.). The pupils were dilated with one drop of 1%, homatropine (*w*/*v*, Humapent-Teva, Debrecen, Hungary). To keep the cornea hydrated and transparent, a drop of artificial tears every 3 min was applied in the experiment. The Bioptigen SD-OCT system (Bioptigen, Research Triangle Park, NC, USA) was used to measure the real-time ocular structure during the eye examination. To investigate the retina, rectangular scans (1000 A-scans × 100 B scans × 3 frames) corresponded to a 3.2 mm × 3.2 mm physical area of the retina. The optic nerve served as the center of the scans. Total and individual retinal thicknesses were determined by the Bioptigen segmentation software InVivoVue Diver 3.3.7.0 at multiple cross-sectional images. The OriginPro 2018 (Macasoft, Gyor, Hungary) statistical package was used for the SD-OCT studies statistical analysis.

### 4.5. Electroretinography (ERG)

Scotopic electroretinography, an essential way to assess retinal function, was performed at day 0, and on the 8th and 16th weeks. Rats were kept under dark adaptation overnight to prepare for scotopic ERG. As Vaczy et al. described [23], under the dim red light (632 nm), rats were anesthetized using the same approach we mentioned above (Section 2.4). Rat was placed on a heating pad throughout the experiment, and its pupils were dilated by one drop of 1% homatropine. ERG recording was conducted with three electrodes. The reference electrode was placed subcutaneously between the eyes, the ground electrode was inserted under the ramp skin, and the surface electrodes applied on the central cornea transduced the electronic potential from the retina responding to light stimuli. The light pulses intensity (5 cd/m^2^, 0.25 Hz, 503 nm green LED light) was preamplified and amplified (2.000×, Bioamp SbA4-V6, Supertech, Pecs, Hungary) and recorded with an A/D converter (Ratsoft-Solar Electronic, Pecs, Hungary). Responses (*n* = 50/eye) were recorded and averaged with Ratsoft software (v.1.0) [23]. Graphs were analyzed further with OriginPro 2018 (Macasoft, Gyor, Hungary). The following parameters were observed: the amplitude and implicit time of the a-wave, b-wave, and oscillatory potentials.

### 4.6. Retina Flat Mount

Rats were terminated after sixteen weeks of STZ treatment, and the dissected eyeballs (4 eyes/control and control + PACAP groups, 10 eyes/diabetes, and 12 eyes/diabetes + PACAP groups) were fixed with 10% buffered formalin (Biosciences, St. Louis, MO, USA) for 2 days. The whole fixed retina was separated from the sclera and rinsed in sterile water (B. Braun, Melsungen, Germany) with mixing, 40–50 mins/time, at least 5–6 times, and then washed overnight. The rinsed retinas were incubated for 7~8 h at 37 °C in 3% trypsin (Gibco™, Thermo Fischer Scientific, Waltham, MA, USA, in 0.1M Tris buffer (pH = 7.8)) with light shaking until retinal tissue started to break into pieces except for vessel network. The separated vessel network was transferred onto a clean slide and dried thoroughly. Samples were stained via PAS/H&E stain, as Jonathan described before [59]. Pericyte ghosts and acellular capillaries were counted under a light microscope (Nikon Eclipse 80i, Auro-Science Consulting Ltd., Budapest, Hungary).

All microvascular histological analysis was based on the relative location of retinal vasculature from the central point of the vessel network, including the peripheral area (between 1500 µm and 3000 µm, 1 picture/quadrant) and edge area (larger than 3000 µm, 1 picture/quadrant). Vessel intensity, the number of pericytes, and acellular capillaries were measured. The vessel intensity was processed and analyzed by NIH ImageJ 1.53k program. The number of pericyte and acellular capillaries were counted in the 1636 × 1088-pixel area and expressed as mean ± SEM.

### 4.7. Data Analysis

Data are expressed as average ± standard error of the mean (SEM) and analyzed using the Kolmogorov–Smirnov normality test. For OGTT, fasting glucose level, C-peptide, ERG (a-wave), OCT (INL and ONL), and analyses of the number of pericytes and acellular capillaries, a nonparametric Kruskal–Wallis’s ANOVA test was used. For triglyceride levels, ERG (b-wave and op-wave), OCT (TRT, IPL, and OS), and vessel density, an ANOVA test followed by Fisher’s LSD post hoc analysis was used. Significant differences were considered at *p*-values below 0.05.

## 5. Conclusions

In conclusion, based on the functional, structural, and microvascular analysis, our study suggested that PACAP38 had a potent protective effect against developing diabetes and proved its power as a therapeutic approach to treat T2DRD in the form of eye drops.

## 6. Limits

This study has a potential limit. The results obtained in the animal model were from male rats. Therefore, the PACAP38 effect in females in T2DRD needs to be considered in the future.

## Data Availability

The original contributions presented in the study are included in the article, further inquiries can be directed to the corresponding author.
